# Symptom network structure of anxiety, depression, and stress among Chinese adolescents: a large-scale cross-sectional study in Fujian Province

**DOI:** 10.3389/fpubh.2026.1959039

**Published:** 2026-10-26

**Authors:** Wenqing Rao, Zhifeng Lin, Hua Jiang, Yixin Wu, Jian Jiang

**Affiliations:** 1 Department of Public Health, Fuzhou Neuropsychiatric Hospital Affiliated to Fujian Medical University, Fuzhou, China; 2 Department of Epidemiology and Health Statistics, School of Public Health, Fujian Medical University, Fuzhou, China; 3 University International College, Macau University of Science and Technology, Macau, Macao SAR, China; 4 Department of Medical Service, Fuzhou University Affiliated Provincial Hospital, Fuzhou, China; 5 Fuzhou University Affiliated Provincial Hospital, Shengli Clinical Medical College of Fujian Medical University, Fujian Emergency Medical Center, Fuzhou, China; 6 School of Health Management, Fujian Medical University, Fuzhou, China

**Keywords:** adolescent, anxiety, depression, network, stress

## Abstract

**Background:**

Anxiety, depression, and stress are highly prevalent mental health problems among adolescents. This study aims to employ network analysis methods to investigate the relationships of anxiety, depression, and stress symptoms among middle and high school students in Fujian, China.

**Methods:**

A total of 57,739 students were included in this study. The Depression Anxiety Stress Scales for Youth (DASS-Y) were used among adolescents. Symptom networks were estimated using regularized Gaussian graphical models with EBICglasso. The stability and precision of the network were assessed by the bootstrap method. Network comparison were performed to exam potential differences by gender, educational stage, and hobby status.

**Results:**

“I hated myself” (Depression5) emerged as the highest mean score symptom. “I felt like I was about to panic” (Anxiety5) and “I found it difficult to relax” (Stress5) were bridge symptoms, showing strong cross-domain connectivity. “I was stressing about lots of things” (Stress3) not only displayed the high centrality in the context of intensity, but also was a bridge symptom. Bootstrap analyses indicated the acceptable precision of edge-weight estimates with and good stability of both strength and bridge strength estimates. Comparison of the networks between adolescents with and without hobby revealed the significant differences in network global strength (absolute difference = 0.38, *p* = 0.048).

**Conclusion:**

Anxiety, depression, and stress symptoms are highly interactive with each other in adolescents. The central and bridge symptoms identified in the network, together with the observed subgroup patterns, provide descriptive evidence and may contribute to future longitudinal and intervention studies.

## Introduction

Adolescent mental disorders have emerged as a major global public health challenge in the 21st century (1). Globally, one in seven adolescents aged 10–19 experiences a mental disorder, with depression and anxiety among the leading causes of illness and disability (2). Adolescence is a rapid developmental period that is greatly influenced by surrounding various factors (3). Adolescents at this stage usually stay at school and face significant academic pressures, interpersonal relationships, and self-identity, often resulting in emotional issues (4). According to Piao et al. (5), over the past 30 years, adolescents have been diagnosed with a variety of mental illnesses, especially anxiety disorders, depressive disorders, accounting for a heavy disease burden on public health.

Meanwhile, a substantial proportion of adolescent experience both anxiety and depression (4). However, the high school and college admissions exams place tremendous pressure on adolescents as well. It is essential to carry out research to promote mental health and prevent mental disorders early among adolescents.

In studies of anxiety and depression, the PHQ-9 (Patient Health Questionnaire-9) and GAD-7 (Generalized Anxiety Disorder-7) scales are widely used (6). The Depression-Anxiety-Stress Scale (DASS) is an internationally recognized self-report psychometric instrument developed by Lovibond (7). This scale provides a quantitative assessment of three negative emotional states, and the total score and subscale scores of the DASS show significant positive correlations with scores from other mental health scales such as the PHQ-9 and GAD-7, demonstrating its good criterion-related validity. Based on it, the research team developed a DASS for Youth (DASS-Y) tailored for Chinese adolescents (8), providing a more detailed assessment of the symptoms underlying co-occurring emotional distress.

In the past, the latent variable model (9) was employed in traditional psychopathological research predominantly, regarding symptoms as mere reflective indicators of underlying diseases, failing to consider the potential interplay among symptoms. Recently, network research has gained substantial attention in psychological sciences (10). In this field of research, psychological behavior is conceptualized as the complex interaction of psychological symptoms and other factors.

Clinically, the joint network is relevant because adolescents often experience overlapping anxiety-, depression-, and stress-related symptoms rather than symptoms confined to a single domain. This pattern is consistent with the tripartite model, which distinguishes shared negative affect from relatively specific anxiety and depression components (11). Analyzing anxiety and depression without considering stress may therefore obscure the contribution of tensional or arousal symptoms and may cause some cross-domain associations to appear to be direct anxiety–depression relationships (11). By modeling all three domains simultaneously, the network can identify symptom connections that may be relevant to the comprehensive assessment and monitoring of co-occurring emotional disorders (12).

Existing network studies have primarily focused on individual symptom domains or on specific symptom dyads, particularly anxiety and depression (13, 14). Within the literature, some studies have also examined whether symptom networks vary across gender (15, 16) and educational stage (16, 17). Although previous work has demonstrated the feasibility of jointly modeling anxiety, depression, and stress symptoms using the DASS-21 (18), evidence remains limited in large, school-based adolescent samples, and little is known about whether the organization of these three symptom domains varies according to hobby status.

The present study addresses these gaps by estimating a symptom network among middle and high school students in Fujian and comparing networks between adolescents with and without a hobby. The analyses were intended to characterize symptom organization and subgroup heterogeneity and to generate hypotheses for future longitudinal and interventional studies.

## Methods

### Study participants

This study is based on the Fujian Adolescents Mental Wellness Study (FAMWeS). FAMWeS is the most extensive study of adolescent mental health in Fujian Province, involving the largest number of schools and the largest sample of students. Using a multistage, stratified, cluster sampling method, a cross-sectional mental health questionnaire survey was conducted among middle and high school students in Fujian Province. The sampling procedure flowchart was displayed in Supplementary Figure S1. Of the 58,174 initially available records, 435 (0.75%) were excluded during data cleaning because of incomplete questionnaires (Supplementary Table S1). The final analytical sample comprised 57,739 participants recruited between April to July 2023. After cleaning and recoding, no missing values remained for the variables used in the characteristic description and network analyses.

### Measures

Homeroom teachers assisted in administering the paper-and-pencil questionnaire in classrooms. This study has been approved by the Ethics Committee of Fuzhou University Affiliated Provincial Hospital (Approval No.: K2023-03-005).

### Sociodemographic data

A self-designed questionnaire was used to collect sociodemographic information from survey participants. The questionnaire covered three aspects: personal background, family circumstances, and academic and school environments. In this study, hobby status was based on participants’ self-reports. A participant was classified as having a hobby if they reported regularly engaging in at least one activity, such as sports, music or smartphone-related activities, at least three times per week, with each session lasting at least 1 h. Participants who did not meet this frequency and duration criterion were classified as having no hobby. Participants were classified into three parenting-style categories: didactic, democratic, and other parenting styles. The didactic style referred to a parenting approach characterized primarily by instruction, advice, or lecturing, whereas the democratic style emphasized dialogue, mutual respect, and adolescent participation in decision-making.

### The depression anxiety stress scales for youth (DASS-Y)

The DASS-Y is a 21-item self-report measure containing three subscales of depression (7 items), anxiety (7 items), and stress (7 items). Participants responded to each item by rating the frequency and severity of symptoms over the past week on a four-point Likert scale from 0 (not true) to 3 (very true). Higher scores indicate severe symptoms in each domain. The reliability and validity of the Chinese version of DASS-Y have been reported in previously published article (8). In this study, the Cronbach’s α value for the DASS-Y was 0.935, while the Cronbach’s α values for the depression, anxiety, and stress subscales were 0.853, 0.827, and 0.840, respectively, indicating good psychometric properties and reliability.

### Data processing

All collected questionnaires were reviewed systematically. Questionnaires with inconsistent responses to repeated trap questions, as well as those with missing, incomplete, or invalid response, were excluded. For questionnaires with complete information, dual data entry was performed using EpiData 3.1 software, followed by consistency checks and logical error correction to ensure data quality. After data entry, a third individual randomly selected 10% of entered data for verification.

### Network estimation

Network estimation was performed using the estimateNetwork() function from the bootnet package. The Gaussian Graphical Model (GGM) using the EBICglasso (qgraph: EBICglasso function()) procedure based on a polychoric correlation matrix was applied to estimate a network of partial correlation coefficients (19). This partial correlation network calculated the parameters between two nodes while controlling for all other variables in the network based on weighted correlation networks.

To reduce spurious connections of the network, the graphical least absolute shrinkage and selection operator (LASSO) was applied for regularization (20). The Extended Bayesian Information Criterion (EBIC) was used to select the optimal model fit, with a default tuning parameter of 0.5. To assess the robustness of the network estimates, the analyses of the EBICglasso parameter tuning = 0.25 and tuning = 0.75 for the network estimation were additionally calculated, respectively. In the network graphs, nodes represent symptoms, and the thickness of edges between nodes reflects the strength of partial correlations. All analyses were performed using R (version 4.3.3) packages qgraph (version 1.9.8) (21).

### Centrality measurement

The network graphs depicted symptoms as nodes, with the thickness of the edges between them serving to indicate the strength of the associations. The quantification of the significant nodes within the network is achieved by the computation of centrality metrics. This study adopted strength as the primary metrics of centrality, which quantifies the total absolute edge weights between a node and its directly connected neighbors, emphasizing the strength of direct connections without intermediaries (22). Nodes with higher strength are regarded as more central to the network. Node centrality was calculated using qgraph:centrality_auto() function and visualized using qgraph:centralityPlot() function.

To identify symptoms that connect distinct symptom communities, bridge strength was calculated by networktools (23) (version 1.5.2) package (bridge() function). Communities were defined *a priori* according to the three DASS-Y subscales: anxiety, depression, and stress. Bridge strength was defined as the sum of the absolute weights of all edges connecting a given node to nodes in the other communities within the same network. Higher bridge strength values therefore indicate stronger cross-community connectivity. The bridge nodes linked the disparate parts of the whole network (24). The stability and accuracy of the network were assessed by the bootstrap method from the bootnet (version 1.5.6) package with bootnet() (19) and corStability() functions. Network precision was assessed using a nonparametric bootstrap with 1,000 resamples. Network stability was assessed separately using a case-dropping bootstrap with 1,000 resamples. The procedure evaluated ten sampling levels across the default range of 5–75% of cases dropped. Correlation stability coefficients were calculated using the corStability() function with the default correlation criterion of 0.70. Besides, the cycle surrounding each node represents its predictability on the network computed by mgm (version 1.2.14) package (25).

### Network comparison

To explore potential differences of the DASS-Y network in gender, educational stage, and hobby, the analyses of the network comparison were performed. A permutation test with 1,000 iterations was used to compute statistical differences in global strength (the absolute sum of all edge weights) and network structure (the distributions of edge weights) across the subgroups, which was calculated by NCT() function from the Network Comparison Test package (version 2.2.2) (26). Subsequently, the Bonferroni-Holm correction was applied to adjust for multiple comparisons. All statistical tests were two-sided, and *p* < 0.050 was considered statistically significant.

## Results

### Characteristics of participants

This study included 57,739 participants in the analysis. The characteristics of the participants are displayed in Table 1. Most participants were middle school student (*n* = 33,687, 58.3%), lived with parents (*n* = 38,797, 67.2%), from complete family (*n* = 51,891, 89.9%). Regarding the Chinese traditional culture, most participants received a didactic parenting style (*n* = 29,885, 51.8%), followed by democratic’s (*n* = 14,139, 24.5%). A total of 44,571 (77.19%) adolescents were classified as having a hobby and 13,168 (22.81%) as having no hobby. The average scores of anxiety symptoms were 3.19 ± 3.82, depression symptoms were 3.22 ± 3.96, and stress symptoms were 5.07 ± 4.37.

**Table 1:** Characteristics of participants.

**Variables**	***N***	**%**
Age (years)	15.00 ± 1.53	
Gender		
Male	29,427	50.97
Female	28,312	49.03
Educational stage		
Middle school	33,687	58.34
High school	24,052	41.66
Region		
Countryside	26,704	46.25
Urban	31,035	53.75
Family		
Incomplete	5,848	10.13
Complete	51,891	89.87
Parenting style		
Didactic	29,885	51.76
Democratic	14,139	24.49
Others	13,715	23.75
Cohabitant		
Parents	38,797	67.19
Father/Mother	10,781	18.67
Others	8,161	14.13
Hobby status		
No	13,168	22.81
Yes	44,571	77.19
Academic performance		
Poor	16,793	29.08
Middle	17,574	30.44
Good	22,793	39.48
Unknown	579	1.00
Establishment of psychological courses		
No	19,320	33.46
Yes	38,419	66.54
Provision of psychological teachers		
No	8,707	15.08
Yes	49,032	84.92
DASS-Y scores		
Anxiety	3.19 ± 3.82	
Depression	3.22 ± 3.96	
Stress	5.07 ± 4.37	

### Symptom scores and node predictability

The participants’ symptom scores were shown in Supplementary Table S2. Stress1 (I got upset about little things) had the highest mean score (1.035 ± 0.844), followed by Stress3 (I was stressing about lots of things) (0.893 ± 0.873), Stress6 (I got annoyed when people interrupted me) (0.674 ± 0.842), and Stress7 (I was easily annoyed) (0.642 ± 0.876). Node predictability in the overall network ranged from 0.353 to 0.602, with a mean of 0.490, indicating that approximately 49.0% of the variance in each symptom was explained by its neighboring nodes on average. Stress7 (I was easily annoyed) showed the highest predictability (0.602), whereas Stress1 (I got upset about little things) showed the lowest (0.353). Detailed node-level predictability values are provided in Supplementary Table S2.

### Overall DASS-Y network estimation

Figure 1 shows the DASS-Y network in the total sample (*n* = 57,739). The network density was 0.70. Of the 147 non-zero edges, 25 (17.01%) were negatively associated, and remaining 112 (82.99%) were positively correlated. The strongest regularized partial association was observed between “Stress4” (I was easily irritated) and “Stress7” (I was easily annoyed) (edge weight = 0.585) within stress communities, “Depression5” (I hated myself) and “Depression6” (I felt like I was no good) (edge weight = 0.487). Moreover, “Depression5” (I hated myself) was weakly negatively associated with “Stress3” (I was stressing about lots of things) (edge weight = −0.073). Table S3 presented the estimated edge weight matrix of the EBICglasso network based on the DASS-Y items.

**Figure 1:**
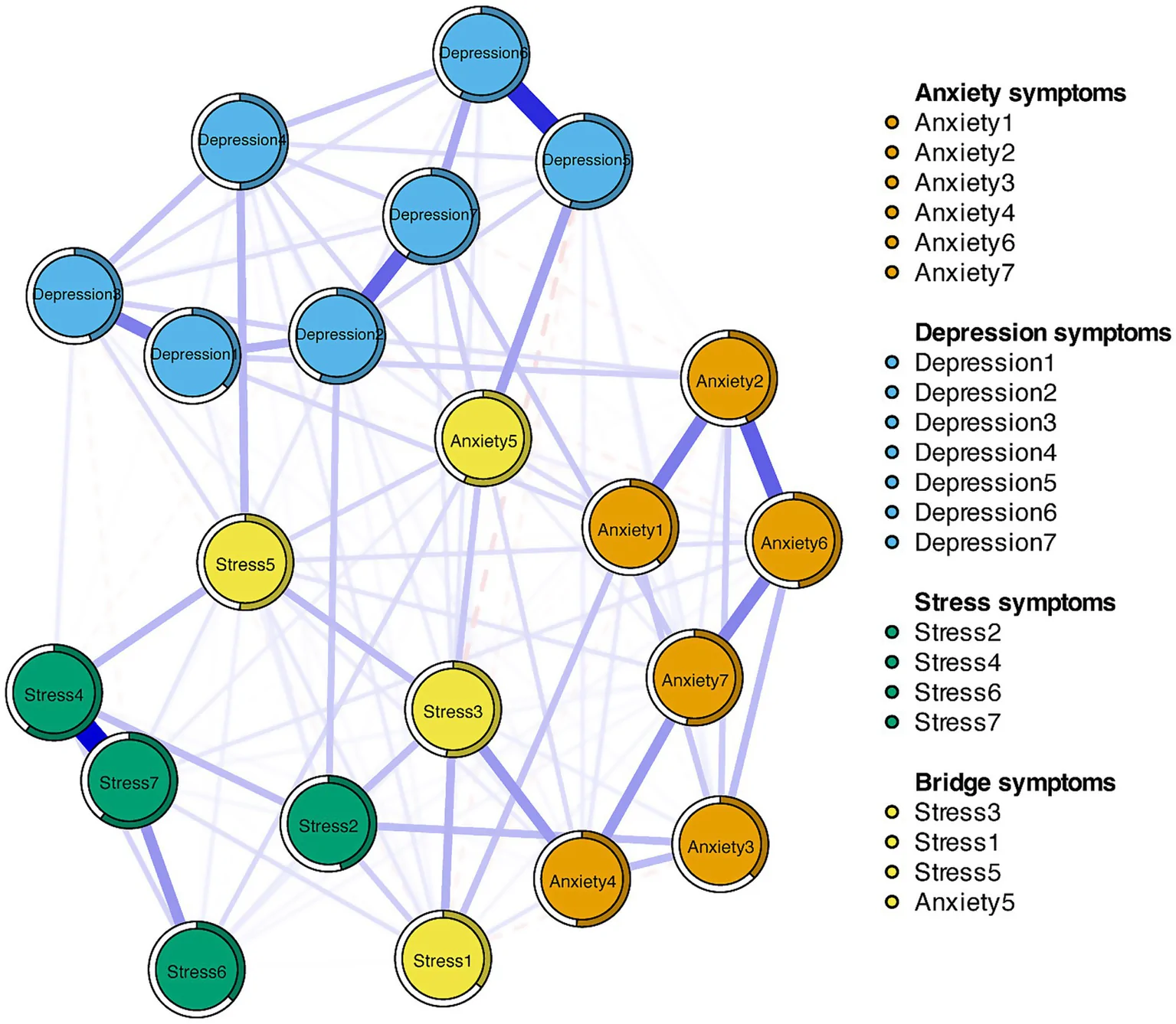
Network structure of DASS-Y.

The centrality plot revealed that “Depression5” (I hated myself) exhibited the prominent centrality, followed by “Stress3” (I was stressing about lots of things) and “Depression2” (I hated my life) (Figure 2A), suggesting that these nodes are the most central positions within the network.

**Figure 2:**
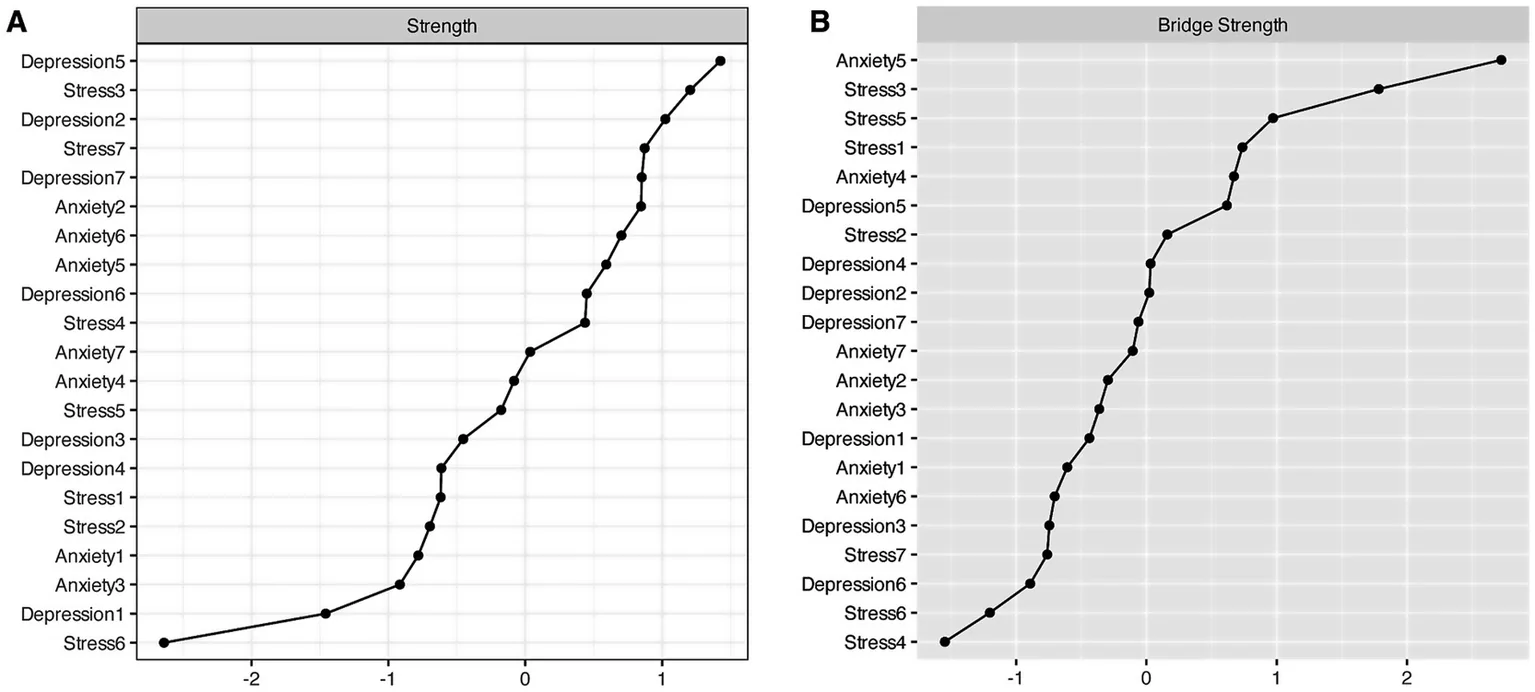
The centrality measures in network. **(A)** Strength, **(B)** Bridge strength.

As demonstrated in Figure 2B and Figure 3, “Anxiety5” (I felt like I was about to panic), “Stress3” (I was stressing about lots of things), “Stress5” (I found it difficult to relax) and “Stress1” (I got upset about little things), exhibited the highest bridge strengths, thereby highlighting them as pivotal bridging symptoms that connect depression, anxiety, and stress problems. In the robustness analyses (Supplementary Figure S2), the main edge weight, centrality and bridge strength patterns were substantively similar in the network, indicating that the principal findings were robust to the choice of the EBICglasso tuning parameter.

**Figure 3:**
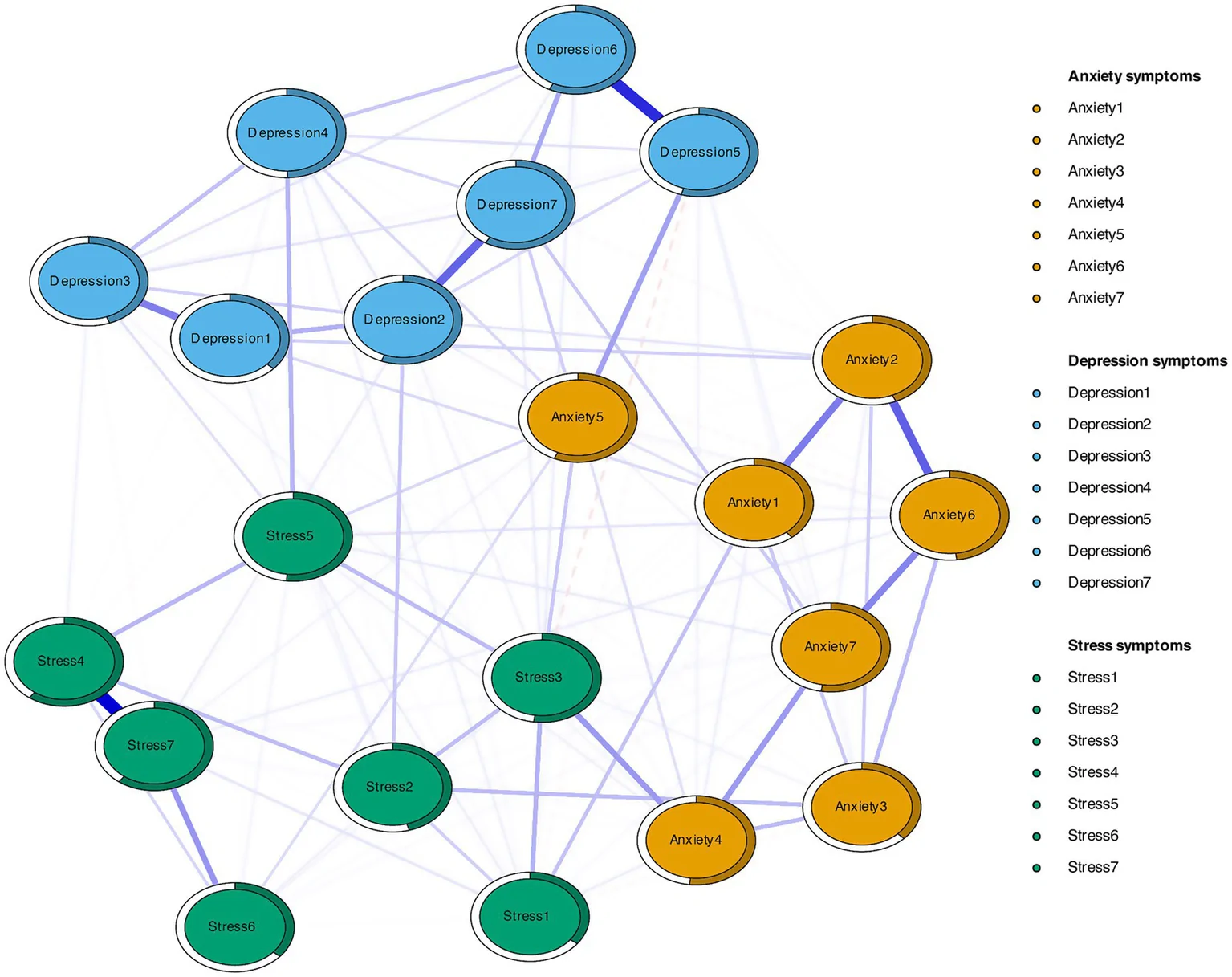
The bridge symptoms in network.

Bootstrap analysis results showed that the bootstrapped confidence intervals are narrow, indicating a high level of precision in the network (Supplementary Figure S3A). The stability coefficient for strength centrality and was 0.75, suggesting that the results of strength centrality was stable (Supplementary Figure S3B). The CS-coefficient for bridge strength centrality was 0.67, indicating acceptable case-dropping stability.

### Subgroup analysis

In the comparison of network models between female and male (Supplementary Figures S4A,B), and middle school and high school (Supplementary Figures S4E,F), there were no significant differences in network global strength (gender: absolute difference = 0.08, *p* = 0.486, Supplementary Figure S4D, educational stage: absolute difference = 0.22, *p* = 0.073, Supplementary Figure S4H), but there was a significant difference in edge weights (gender: maximum absolute edge-weight difference = 0.08, *p* < 0.001, Supplementary Figure S4C, educational stage: maximum absolute edge-weight difference = 0.08, *p* < 0.001, Supplementary Figure S4G). Additionally, there were significant differences in network global strength (absolute difference = 0.38, *p* = 0.048, Figure 4D), but not in edge weights (maximum absolute edge-weight difference = 0.05, *p* = 0.364, Figures 4A–C) between hobby and non-hobby.

**Figure 4:**
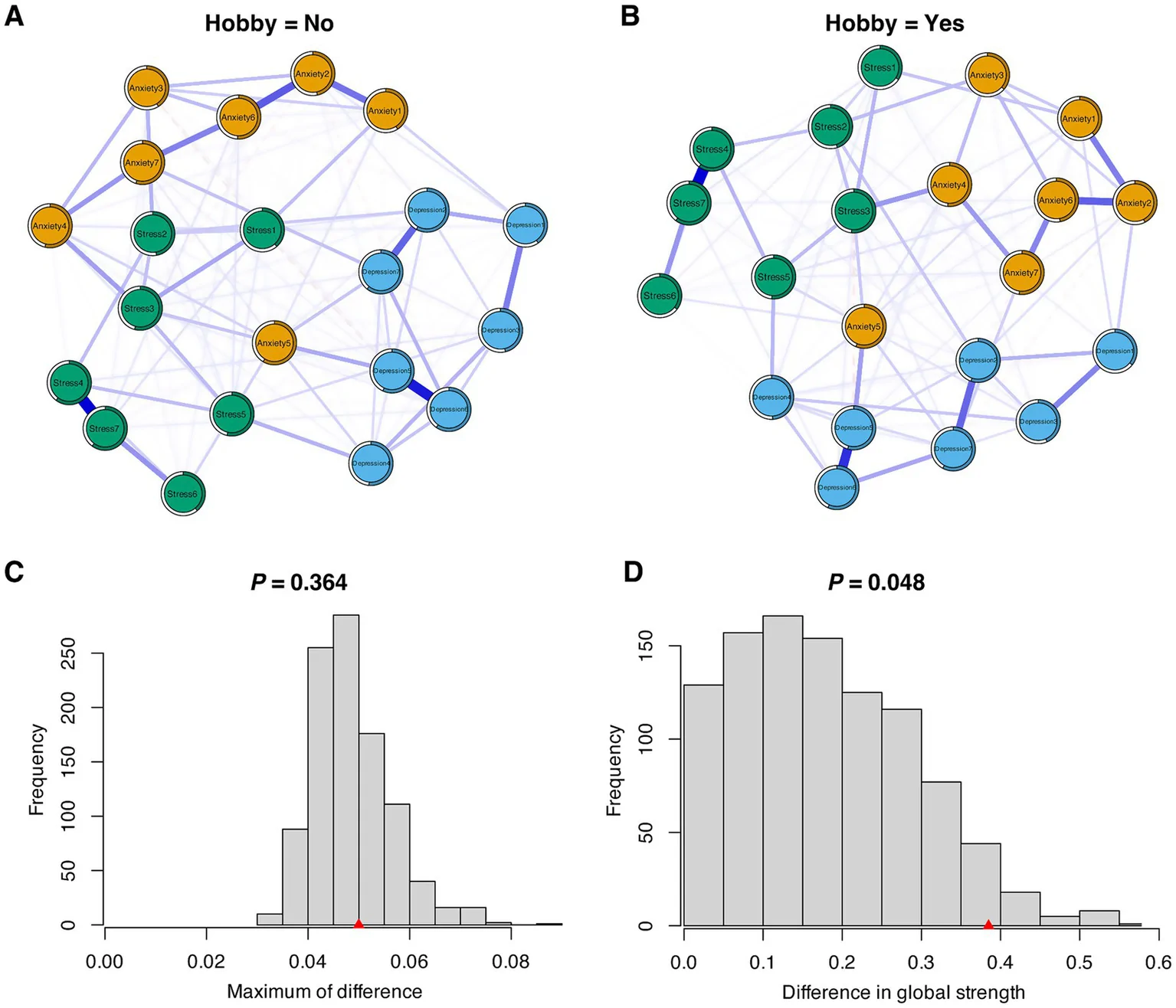
The network comparison between non-hobby **(A)** and hobby **(B)**. **(C)** The difference in edge weights. **(D)** The difference in network global strength.

## Discussion

In this study, we conducted a network analysis to explore the network structures of symptoms among depression, anxiety, and stress in adolescents. We identified several symptoms with the high centrality and bridge strength in the network. First, “I got upset about little things” (Stress1) emerged as the symptom with the highest mean score. This finding may reflect difficulties in emotional regulation during adolescence and the distinctiveness of mental health concerns in this age group. This symptom is not an isolated pathological phenomenon, but rather one of the core manifestations of various psychological disorders. “Irritability and anger” were common symptoms in anxiety disorders (27). Besides, “Persistent sad or irritable mood” was a sign of severe depression in adolescents according to the World Health Organization (28). When referring to their emotions in adolescents, they frequently described using “easily irritable,” “snappy” terms (29).

Second, “I was stressing about lots of things” (Stress3) not only displayed the high centrality in the context of intensity, but also was a bridge symptom, suggesting that this symptom may be relevant to the observed comorbid network of depression, anxiety, and stress. In cross-cultural clinical samples, stress nodes were the most likely bridge symptoms between depressive and anxiety symptoms (30), this pattern also observed in the present adolescent sample. Moreover, the phrase “lots of things” may reflect a broad and multifaceted perception of stress. It could be hypothesized that this broad perception might make it difficult to identify a single source of stress; however, this interpretation requires direct evaluation in future longitudinal studies.

This diffuse sense of pressure was prone to being intertwined with other negative emotions. In addition, this feeling subsequently constituted the fundamental substance of rumination (31). Adolescents may repeatedly contemplate questions such as “Why is there so much pressure on me?” or “What actions should I take?”. These cognitive patterns have been associated with depression and anxiety, thereby providing context for the association between this node and symptoms of depression and anxiety.

Furthermore, “I felt like I was about to panic” (Anxiety5) and “I found it difficult to relax” (Stress5) were other bridge symptoms in the DASS-Y network, suggesting that these symptoms are associated with spanning symptom communities among depression, anxiety, and stress. Multiple studies showed that “difficulty relaxing,” as a persistent state of physiological and psychological hyperarousal, was one of the most significant bridge symptoms in the network of depression and anxiety among adolescents (32, 33).

“I felt like I was about to panic” (Anxiety5) describes an acute, catastrophic outburst of physiological responses. Unlike the more persistent experience of “difficulty relaxing” panic represents a more acute anxiety-related experience. This intense, sudden sense of loss of control and impending doom was associated with depressive symptoms. The bridging symptoms may exhibit network properties consistent with potentially relevant correlates in the development of comorbid conditions. For instance, adolescents may first experience prolonged “difficulty relaxing” and academic stress (34). However, the cross-sectional design does not allow to determine the direction or temporal ordering of these associations.

The bridge symptoms showed conditional associations connecting the anxiety, depression, and stress symptoms. For example, difficulty relaxing was associated with depressive symptoms such as fatigue, anhedonia, and worthlessness, and these symptoms co-occurred in the estimated network. Clinically, bridge symptoms may be candidates for trans-diagnostic interventional research, whether targeting them improves symptoms across domains requires prospective evaluation.

In examining the network structures between hobby and non-hobby, the results revealed significant differences in network global strength but not in edge weights. Although the present study compared adolescents only according to whether they reported having a hobby, rather than distinguishing among specific types of hobbies, the observed difference in global network strength provides a preliminary clue that hobby-related characteristics may be relevant to the interactions of emotional symptoms. For example, Aumer et al. showed that physical activity had a good treatment effect on depressive symptoms in adolescents (35). Besides, the interventions of bibliotherapy (36), art therapy (37) were strong evidence on the improvement of anxiety and depression in adolescents. The more fine-grained longitudinal or interventional researches are needed to determine whether particular types of hobbies are associated with changes in adolescent symptom networks.

## Limitations

There are several limitations in this study. First, the cross-sectional design precludes causal inferences regarding the directionality of associations among symptoms. Additionally, because participating schools may differ from other schools in unmeasured characteristics, the representativeness and generalizability of the findings may be limited. Another limitation concerns the clustered sampling structure. Students were sampled within classes and schools, but the network analyses treated individual observations as independent and did not explicitly account for class- or school-level clustering. Consequently, future studies should apply multilevel or cluster-adjusted network methods to distinguish individual-level symptom associations. Second, data were collected via self-report questionnaires, which are subject to recall and social desirability biases. Excluding incomplete questionnaires may have introduced selection bias. In addition, the binary operationalization of hobby status did not capture hobby type, frequency, intensity, or personal meaning, limiting interpretation of the hobby-related network differences. Third, although the sample was drawn from six prefectures in Fujian Province, the findings may not generalize to adolescents in other Chinese regions or cultural contexts, particularly those with different educational systems or socioeconomic profiles. Fourth, the EBICglasso regularization method, while effective at reducing false positive edges, may still produce unstable edge estimates under certain conditions. Given the large sample size, the subgroup comparisons had high statistical power, such that small deviations in edge weights could reach statistical significance. Therefore, the statistically significant edge-level findings should be interpreted alongside their effect magnitudes and should not be assumed to indicate substantial practical differences. Similarly, although the bootstrap confidence intervals were narrow, they indicate relatively precise estimates within this sample and do not, by themselves, establish practical importance or model validity. Fifth, the network model inherently assumes no unmeasured confounding—a strong assumption that cannot be fully verified with cross-sectional data. Sixth, the DASS-Y comprises only 21 items and may not capture other clinically relevant symptom dimensions such as sleep disturbances, social anxiety, or non-suicidal self-injury, which may interact with the symptoms examined in this study.

## Conclusion

This study investigated the network of anxiety, depression, and stress symptoms among adolescents and examined whether it differed according to hobby status. The identified central and bridge symptoms may inform hypotheses for future researches. These findings describe the conditional association structure of co-occurring emotional symptoms. Longitudinal studies are needed to examine the temporal evolution of symptom networks and the relationship between hobby status and symptom dynamics.

## Data Availability

The datasets presented in this article are not readily available because the datasets during the current study available from the corresponding author on reasonable request. The data are not publicly available due to privacy or ethical restrictions. Requests to access the datasets should be directed to Jian Jiang, sx0191@fzu.edu.cn.
