# Supplementary material for: Symptom network structure of anxiety, depression, and stress among Chinese adolescents: a large-scale cross-sectional study in Fujian Province
Source: Front Public Health. 2026 Oct 26;14:1959039. doi: 10.3389/fpubh.2026.1959039 (PMC13745259; doi:10.3389/fpubh.2026.1959039)
Supplement: Supplementary file 1 [file Supplementary_file_1.DOCX]

**Table S1. Comparison of participants excluded during data cleaning and those included in the analytical sample**

| **Variable** | **Category or summary** | **Excluded (n = 435)** | **Included (n = 57,739)** | **P value** |
| --- | --- | --- | --- | --- |
| **Age** | Mean (SD) | 14.70 (1.58) | 15.00 (1.53) | <0.001 |
| **Total DASS-Y score** | Mean (SD) | 9.49 (10.33) | 11.48 (11.01) | <0.001 |
| **Depression score** | Mean (SD) | 2.57 (3.38) | 3.22 (3.96) | <0.001 |
| **Anxiety score** | Mean (SD) | 2.60 (3.76) | 3.19 (3.82) | 0.001 |
| **Stress score** | Mean (SD) | 4.32 (4.31) | 5.07 (4.37) | <0.001 |
| **Gender** | Male | 259 (59.5%) | 29427 (51.0%) | <0.001 |
|  | Female | 176 (40.5%) | 28312 (49.0%) | <0.001 |
|  | Missing | 0 (0.0%) | 0 (0.0%) | <0.001 |
| **Educational stage** | Middle school | 285 (65.5%) | 33687 (58.3%) | 0.002 |
|  | High school | 150 (34.5%) | 24052 (41.7%) | 0.002 |
|  | Missing | 0 (0.0%) | 0 (0.0%) | 0.002 |
| **Region** | Countryside | 168 (38.6%) | 26704 (46.2%) | 0.001 |
|  | Urban | 267 (61.4%) | 31035 (53.8%) | 0.001 |
|  | Missing | 0 (0.0%) | 0 (0.0%) | 0.001 |
| **Hobby status** | No | 111 (25.5%) | 13168 (22.8%) | 0.002 |
|  | Yes | 323 (74.3%) | 44571 (77.2%) | 0.002 |
|  | Missing | 1 (0.2%) | 0 (0.0%) | 0.002 |
| **Family** | Complete | 332 (76.3%) | 51891 (89.9%) | <0.001 |
|  | Incomplete | 31 (7.1%) | 5848 (10.1%) | <0.001 |
|  | Missing | 72 (16.6%) | 0 (0.0%) | <0.001 |
| **Parenting style** | Didactic | 124 (28.5%) | 29885 (51.8%) | <0.001 |
|  | Democratic | 67 (15.4%) | 14139 (24.5%) | <0.001 |
|  | Other | 58 (13.3%) | 13715 (23.8%) | <0.001 |
|  | Missing | 186 (42.8%) | 0 (0.0%) | <0.001 |
| **Cohabitant** | Parents | 173 (39.8%) | 38797 (67.2%) | <0.001 |
|  | Father or mother | 51 (11.7%) | 10781 (18.7%) | <0.001 |
|  | Others | 32 (7.4%) | 8161 (14.1%) | <0.001 |
|  | Missing | 179 (41.1%) | 0 (0.0%) | <0.001 |
| **Academic performance** | Good | 163 (37.5%) | 22793 (39.5%) | <0.001 |
|  | Moderate | 136 (31.3%) | 17574 (30.4%) | <0.001 |
|  | Poor | 116 (26.7%) | 16793 (29.1%) | <0.001 |
|  | Unknown or missing | 20 (4.6%) | 579 (1.0%) | <0.001 |
| **Establishment of psychological courses** | Yes | 301 (69.2%) | 38419 (66.5%) | <0.001 |
|  | No | 130 (29.9%) | 19320 (33.5%) | <0.001 |
|  | Missing | 4 (0.9%) | 0 (0.0%) | <0.001 |
| **Provision of psychological teachers** | Yes | 381 (87.6%) | 49032 (84.9%) | <0.001 |
|  | No | 50 (11.5%) | 8707 (15.1%) | <0.001 |
|  | Missing | 4 (0.9%) | 0 (0.0%) | <0.001 |

Note: Continuous variables are presented as mean (SD), and categorical variables as n (%). P values for continuous variables were obtained using Welch's t-test; categorical variables were compared using Fisher's exact test.
