# Supplementary material for: Symptom network structure of anxiety, depression, and stress among Chinese adolescents: a large-scale cross-sectional study in Fujian Province
Source: Front Public Health. 2026 Oct 26;14:1959039. doi: 10.3389/fpubh.2026.1959039 (PMC13745259; doi:10.3389/fpubh.2026.1959039)
Supplement: Supplementary file 2 [file Supplementary_file_2.DOCX]

**Table S2. Symptom scores and node predictability of participants (n = 57,739)**

| **Variable** | **Items context** | **Mean** | **SD** | **IQR** | **Skewness** | **Kurtosis** | **Predictability** |
| --- | --- | --- | --- | --- | --- | --- | --- |
| **Stress1** | I got upset about little things | 1.035 | 0.844 | 0-1 | 0.690 | 0.072 | 0.353 |
| **Anxiety1** | I felt dizzy, like I was about to faint | 0.505 | 0.737 | 0-1 | 1.518 | 2.014 | 0.390 |
| **Depression1** | I did not enjoy anything | 0.508 | 0.714 | 0-1 | 1.428 | 1.838 | 0.368 |
| **Anxiety2** | I had trouble breathing (e.g. fast breathing), even though I wasn't exercising and I was not sick. | 0.331 | 0.652 | 0-0 | 2.204 | 4.846 | 0.435 |
| **Depression2** | I hated my life | 0.463 | 0.717 | 0-1 | 1.665 | 2.621 | 0.560 |
| **Stress2** | I found myself over-reacting to situations | 0.572 | 0.761 | 0-1 | 1.324 | 1.382 | 0.455 |
| **Anxiety3** | My hands felt shaky | 0.430 | 0.72 | 0-1 | 1.806 | 2.984 | 0.374 |
| **Stress3** | I was stressing about lots of things | 0.893 | 0.873 | 0-1 | 0.782 | -0.063 | 0.523 |
| **Anxiety4** | I felt terrified | 0.575 | 0.779 | 0-1 | 1.366 | 1.444 | 0.519 |
| **Depression3** | There was nothing nice I could look forward to | 0.535 | 0.802 | 0-1 | 1.504 | 1.616 | 0.443 |
| **Stress4** | I was easily irritated | 0.638 | 0.846 | 0-1 | 1.282 | 0.940 | 0.600 |
| **Stress5** | I found it difficult to relax | 0.613 | 0.824 | 0-1 | 1.301 | 1.036 | 0.519 |
| **Depression4** | I could not stop feeling sad | 0.473 | 0.753 | 0-1 | 1.657 | 2.271 | 0.498 |
| **Stress6** | I got annoyed when people interrupted me | 0.674 | 0.842 | 0-1 | 1.200 | 0.811 | 0.363 |
| **Anxiety5** | I felt like I was about to panic | 0.493 | 0.780 | 0-1 | 1.652 | 2.190 | 0.566 |
| **Depression5** | I hated myself | 0.315 | 0.668 | 0-0 | 2.371 | 5.454 | 0.549 |
| **Depression6** | I felt like I was no good | 0.452 | 0.744 | 0-1 | 1.750 | 2.653 | 0.574 |
| **Stress7** | I was easily annoyed | 0.642 | 0.876 | 0-1 | 1.306 | 0.871 | 0.602 |
| **Anxiety6** | I could feel my heart beating really fast, even though I hadn't done any hard exercise | 0.396 | 0.715 | 0-1 | 1.973 | 3.594 | 0.481 |
| **Anxiety7** | I felt scared for no good reason | 0.465 | 0.765 | 0-1 | 1.730 | 2.475 | 0.527 |
| **Depression7** | I felt that life was terrible | 0.475 | 0.766 | 0-1 | 1.699 | 2.399 | 0.586 |
