# Supplementary material for: Symptom network structure of anxiety, depression, and stress among Chinese adolescents: a large-scale cross-sectional study in Fujian Province
Source: Front Public Health. 2026 Oct 26;14:1959039. doi: 10.3389/fpubh.2026.1959039 (PMC13745259; doi:10.3389/fpubh.2026.1959039)
Supplement: Supplementary file 3 [file Supplementary_file_3.DOCX]

**Supplemental Figures**

Figure S1. The sampling procedure flowchart in this study.

Figure S2. Robustness analyses using alternative EBICglasso tuning parameters. A–C: Strength, bridge strength, and symptom network estimated with tuning = 0.25; D–F: the corresponding estimates with tuning = 0.75.

Figure S3. Bootstrap analysis results for node and bridge strength. A: plot of the bootstrapped confidential intervals; B: The stability coefficient for strength centrality.

Figure S4. The comparison of networks. A-D: the comparison of network models between female and male; E-H: the comparison of network models between middle school and high school.
